# Controlled Ligand-Free Growth of Free-Standing CsPbBr_3_ Perovskite Nanowires

**DOI:** 10.1021/acsomega.4c06646

**Published:** 2024-11-26

**Authors:** Ziyun Huang, Zhaojun Zhang, Nils Lamers, Dmitry Baranov, Jesper Wallentin

**Affiliations:** 1 Synchrotron Radiation Research and NanoLund, Department of Physics, Lund University, Box 124, Lund 22100, Sweden; 2 Division of Chemical Physics and NanoLund, Department of Chemistry, Lund University, Box 124, Lund 22100, Sweden

## Abstract

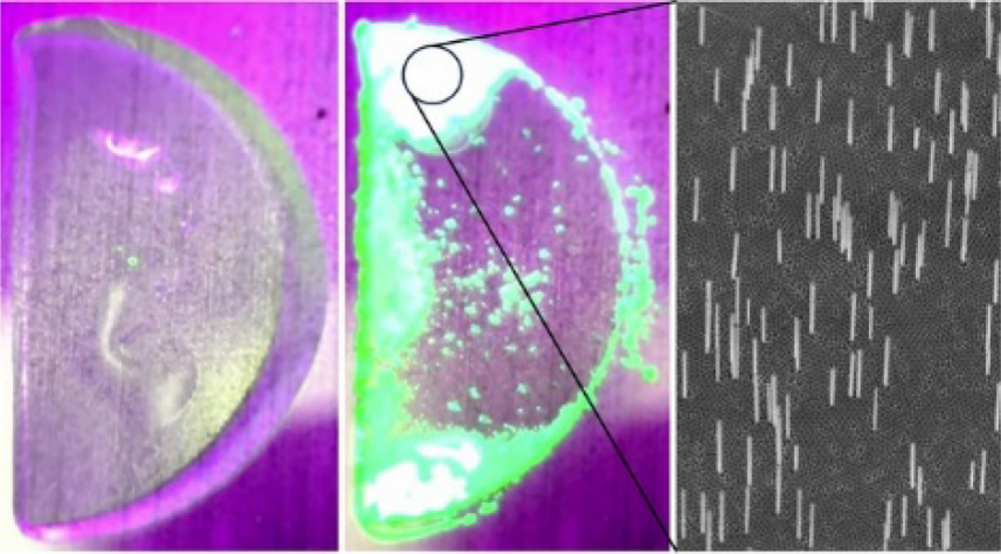

Metal halide perovskite nanowires are widely studied due to their unique electronic and optical characteristics, making them promising for light emitting and detection applications. We developed a ligand-free method to grow vertically aligned free-standing CsPbBr_3_ nanowires from anodized aluminum oxide nanopore substrates. Here, we investigate the growth process using *in situ* microscopy with ultraviolet and visible light excitation, revealing a highly dynamic process with pronounced fluorescence at locations where high-density free-standing nanowires could be found. The yield of the growth is strongly improved by using a growth reactor with controlled N_2_ flow, increasing from 17 to 60%. We systematically investigated the growth dependence on the temperature and N_2_ flow rate and identified optimal parameters at 70 °C and 0.8 L/min, respectively. The improved control over the growth of free-standing nanowires expands opportunities for their integration into optoelectronic devices.

## Introduction

Metal halide perovskite (MHP) nanowires have made remarkable progress recently in various fields due to their excellent optoelectronic and photovoltaic properties and low-cost fabrication, offering promising applications in lasers, light emitting diodes (LEDs), solar cells, photodetectors, and X-ray scintillators.^1−9^ The synthesis of MHP nanowires has been widely studied, and various growth methods have been reported and mechanisms proposed.^10^ The solution-based growth methods are inexpensive, simple, and easily scalable. A commonly used solution-based approach for MHP nanowire synthesis is the colloidal synthetic method, which utilizes organic ligands as stabilizers to control the size and morphology of the nanowires.^11,12^ However, a significant limitation of this method is its lack of control over nanowire orientation and positioning, posing challenges for integration into devices such as LEDs and solar cells.^11−13^ The dissolution–recrystallization process is another solution-based method that converts metal sources into perovskites by dipping the metal source thin film (e.g., lead halide or lead acetate) into halide salts such as CsBr or CH_3_NH_3_Br.^5,14−18^ This method allows nanowires to grow directly on top of an electrode, simplifying the device fabrication process. However, issues with controlling the orientation and morphology of the nanowires persist.^7,17,19,20^ A more controlled growth process widely used in synthesis is the chemical vapor deposition (CVD) method, which evaporates, sputters, or deposits chemicals directly onto the substrate.^21,22^ Moreover, orientational control can be achieved by using the epitaxial relationship between the perovskite lattice and substrates.^23−25^ CVD produces high-quality nanowires with high crystallinity and low defects, but it is a complex procedure that requires high vacuum and high temperature compared with solution growth methods, making it unsuitable for most hybrid perovskites.^25−27^ Vapor–liquid–solid (VLS) synthesis, which incorporates a liquid catalyst compared with the CVD process, is another growth method that produces highly crystalline nanowires with precise control over chemical composition and nanowire density.^28,29^ Similar to CVD, VLS synthesis requires both high temperatures and vacuum conditions and often results in residual catalyst material remaining on the nanowire tip postsynthesis.^30^

To achieve control over the morphology and orientation of nanowire arrays in atmosphere and low temperature, templates were introduced to the solution growth process of MHP nanowires.^31−33^ Among other templates, anodized aluminum oxide (AAO) templates^34−36^ with vertically oriented nanopores stand out for several reasons:^19^ (i) The AAO nanopore structure can be prepared directly on a conductive substrate, which is advantageous for device integration, (ii) the diameter and spacing of AAO nanopores can be easily tuned, which helps control the diameter of nanowires grown from the pores,^19,37^ and (iii) the vertical orientation is ideal for devices with light emission and absorption orthogonal to the substrate. Therefore, several groups have demonstrated growth of MHP nanowires inside AAO templates.^19^

We have recently shown that AAO templates can also be used to synthesize free-standing nanowires, which surprisingly continue to grow outside of the AAO template.^38^ The method is ligand-free and based on simple solution-based low-temperature crystallization. The nanowires can subsequently be used for blue-green heterostructured nanowires^38−40^ and single-nanowire devices.^41^ However, the yield of the initially reported method was low, and multiple attempts were normally required to produce a sample with a satisfactory number of free-standing nanowires. Here, we show that drastically improved yield and repeatability can be achieved by controlling the evaporation rate. We accomplished that by using a laminar flow growth reactor with a controlled N_2_ flow instead of direct evaporation in a fume hood, which has been used previously. First, *in situ* microscopy was used to track the growth of nanowires and provide a better understanding of the growth process. It was found that locations where nanowires were observed exhibited pronounced fluorescence during the initial stages of growth, attributed to the initial crystallization of the MHP. Then, the optimal crystallization conditions were identified through systematic variation of temperature and flow rate. We observe a relatively large growth window at temperatures between 50 and 80 °C and flow rates between 0.8 and 1.0 L/min. The most favorable growth parameters were identified as 70 °C with a flow rate of 0.8 L/min. Free-standing nanowires could be found on 60% of the AAO templates when grown under the best growth conditions, in contrast to only 17% without the growth chamber.

## Methods

**Figure 1:**
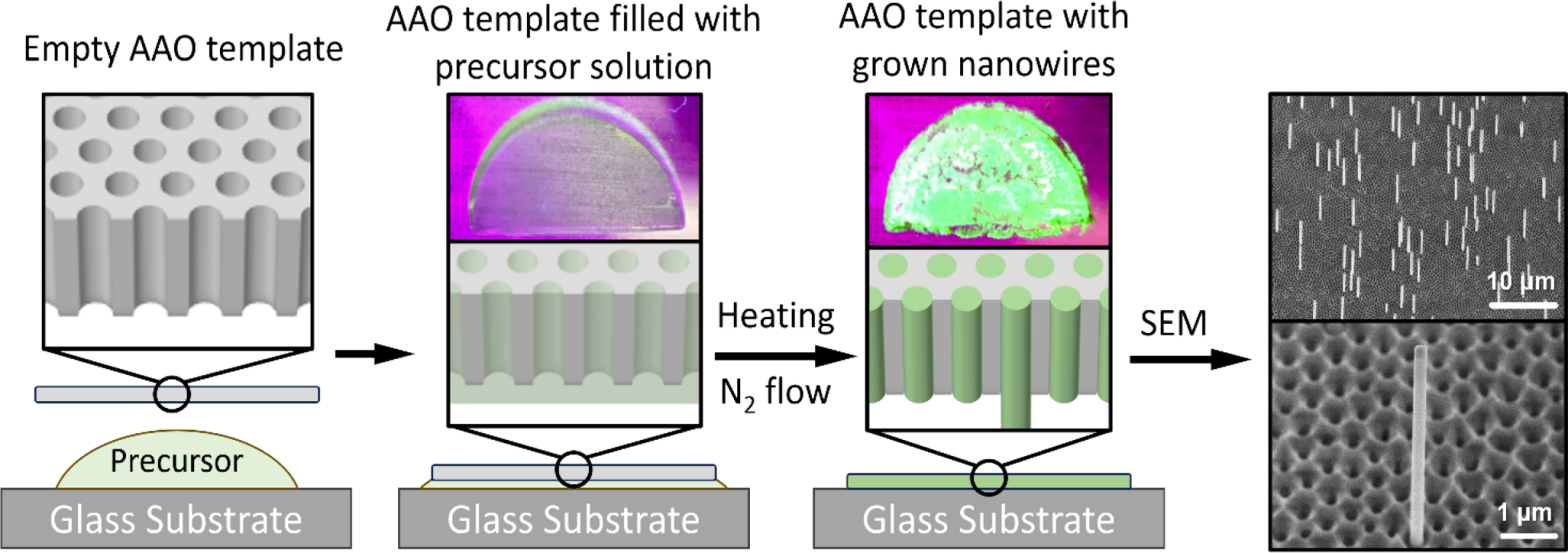
Schematic diagram of the growth process.

The growth process is shown in Figure 1. First, 20 μL of precursor solution (0.3 M CsPbBr_3_), which is an equimolar mixture of CsBr (99.9%, Thermo Scientific) and PbBr_2_ (>98.0%, TCI) in DMSO (≥99.5%, max. 0.03% H_2_O, VWR or Supelco), was dropped on a clean glass substrate by a mechanical micropipette. Then, a commercial AAO template manufactured by Shenzhen Topmembranes Technology Co., Ltd. with a 170 nm pore diameter was put on top of the droplet such that it floated on top of the precursor solution. The AAO template was infiltrated with the precursor solution due to capillary forces. The whole sample was then transferred to the growth chamber, which is a rectangular aluminum tube, with the top side replaced by transparent glass. The tube dimensions are 2.15 cm wide, 1.1 cm tall, and 15 cm long, with an N_2_ gas source connected to one end of the chamber and a hot plate placed underneath. A schematic of the setup is shown in Figure S1. The N_2_ gas flow rate through the chamber is controlled by a gas flow meter, and the hot plate heat keeps the chamber at a desired temperature. The sample was kept in the growth chamber for 12 h, during which the solvent evaporated from the precursor solution, leading to crystallization and the formation of vertically aligned free-standing CsPbBr_3_ nanowires on the backside of the AAO template. The growth process was recorded using a Digital Portable USB microscope 1600X connected to a computer. The white light illumination was provided by the LED source of the microscope, and a UV torch (365 nm) was used for occasional UV illumination to observe photoluminescence. The samples were imaged by SEM (scanning electron microscopy), and the area of the regions with nanowires was obtained and calculated using the ImageJ software.^42^ The growth area ratio, which is later used as a parameter for quantifying the growth conditions, is the total area that contains nanowires in a single AAO sample divided by the area of the AAO template used. The growth area ratio should not be confused with the density of the nanowires.

## Results and Discussion

In our previous research, it was observed that the nanowires first crystallize and grow inside the AAO cylindrical nanopores.^38^ Most AAO nanopores are filled with single-crystal nanowires that grow in the ⟨001⟩ direction, and some of them continue to grow outside and form free-standing nanowires. The free-standing nanowires exhibit (110)-type side facets, showing that this is the lowest-energy facet. However, the dynamics of the growth of free-standing nanowires were poorly understood.

**Figure 2:**
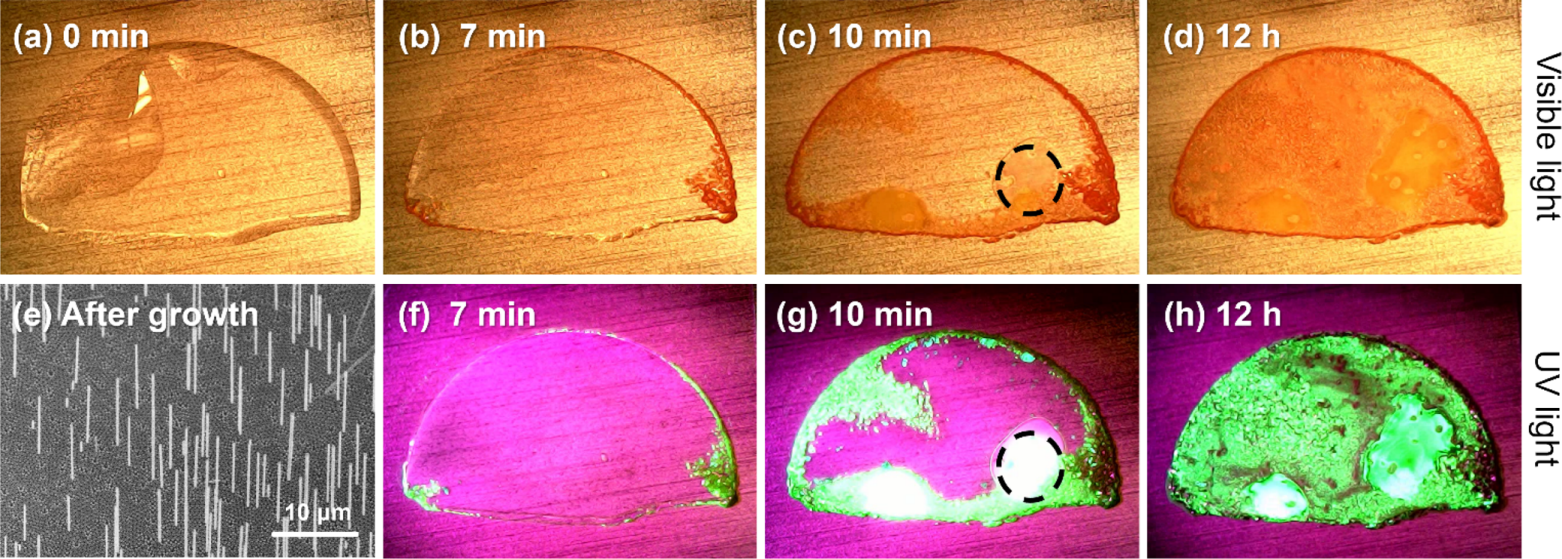
*In situ* microscopy. Snapshots of the video during the growth process. Images of the sample (a) at the beginning of growth, (b) after 7 min of growth, (c) after 10 min of growth, and (d) after 12 h of growth. (e) SEM image of nanowires on AAO after growth. This image was taken at the place circled in panels (c, g), where the light excitation effect is strongest under UV light. Images of the sample under UV light (f) after 7 min of growth, (g) after 10 min of growth, and (h) after 12 h of growth.

To get more insight into the growth process, we used *in situ* microscopy. The free-standing nanowires grow between the glass substrate and the AAO template, but thanks to the transparency of the AAO membranes and the precursor solution, we could still observe the growth process at the beginning of crystallization. The video was recorded with white light illumination for most of the growth time with occasional UV illumination at selected stages. After the growth, we checked which areas resulted in free-standing nanowires using SEM. Snapshots of the video under visible light are shown in Figure 2a–d, and snapshots under UV light are shown in Figure 2f–h.

Figure 2a illustrates the semicircular AAO template floating on the CsPbBr_3_ precursor solution at the onset of the growth process. After 7 min of heating, crystallization begins, as shown in Figure 2b,f, where crystals emitting green light under UV illumination are observed at the edge of the AAO template. Once the heating process was initiated, the solvent began to evaporate, leading to a supersaturation of the solution and crystal growth. The solvent between the AAO template and the glass substrate can evaporate either from the edge of the AAO template or through the AAO nanopores. We observe that supersaturation first occurs at the template’s edge, as seen in Figure 2b,f, likely due to its direct exposure to the flowing N_2_ gas.

After 10 min, a bubble spontaneously formed near the edge of the AAO template, as highlighted in Figure 2c,g by the black circle, and that was quickly followed by crystallization manifested as bright green photoluminescence within the bubble area. By correlating the microscopy images with ex situ SEM, we identified these regions as the primary sites for the growth of free-standing nanowires. The black circle in Figure 2c,g marks the area where most nanowires were found, which is also where the SEM image shown in Figure 2e was taken. Figure 2d,h shows the fully crystallized AAO template after 12 h of continuous heating. From these results, we can conclude that the nanowires grew in the regions where the N_2_ bubble was formed. We have observed the same type of process in more than five growth runs.

Based on the above observations, we can make a hypothesis for the free-standing nanowire growth process. The formation of a N_2_ bubble creates a new solution-N_2_ interface, which significantly speeds up the solvent evaporation locally. The precursor solution inside the AAO nanopores near the N_2_ bubble rapidly evaporates and reaches supersaturation, leading to heterogeneous nucleation inside the AAO nanopores.^43^ The nuclei further crystallize into nanowires within the area characterized by strong photoluminescence under UV light, as shown in Figure 2g. Once the nanowires reach the bottom opening of the AAO nanopores, the (001) bottom facets act as the substrates for further growth. Given that the (001) facets have a higher solid–liquid interfacial energy than the (110) facets, the nanowires continue to grow along the ⟨001⟩ direction until the precursor solution is fully consumed. Figure 2e shows that the AAO region with free-standing nanowires correlates with the enhanced photoluminescence under UV illumination at the beginning of the growth process. We tentatively suggest that the increased photoluminescence is indicative of nuclei responsible for high yield of free-standing nanowires (for example, nuclei of specific morphology or location in the AAO membrane).

We hypothesize that the N_2_ bubble forms due to mechanical forces, although the exact mechanism for this is not clear. We note that the 15-μm thick AAO membrane has some flexibility. The bubble forms after observable crystallization around the edge, which partially seals the edge of the AAO template and reduces the evaporation. The crystals formed at the edges could also attach the membrane to the substrate. One possibility is that the edge crystallization bends the membrane since the CsPbBr_3_ crystal volume is only about 10% of the corresponding precursor volume.^43^ This bending could lead to bubble formation.

**Figure 3:**
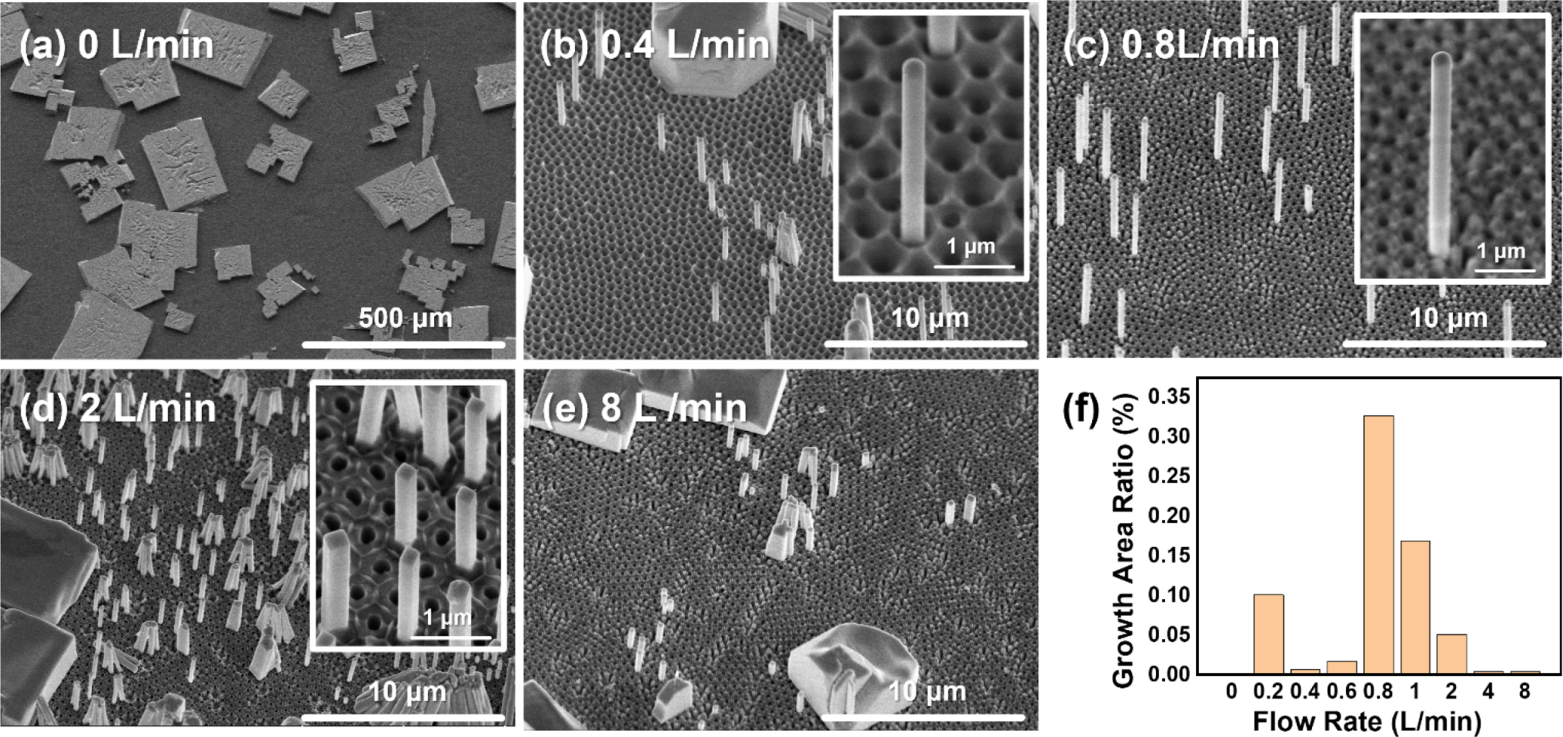
Flow rate variation. SEM images of free-standing nanowires grew at gas flow rates of (a) 0 L/min, (b) 0.4 L/min, (c) 0.8 L/min, (d) 2 L/min, and (e) 8 L/min. The inset shows the high-resolution SEM image of single/several nanowires at each gas flow rate. (f) Growth area ratio at different flow rates. The insets show high-resolution SEM images of single nanowires at each flow rate. The SEM images are tilted by 30°.

Using our initially reported growth method,^38^ performed directly in a fume hood, only approximately 17% (3 out of 18 according to our latest experiment) of the templates produced nanowires due to a rather uncontrolled evaporation. We therefore developed a laminar flow growth reactor where the N_2_ flow could be controlled. We then optimized the growth conditions by systematically varying the N_2_ flow and temperature. First, nanowires were grown under different flow rates ranging from 0 to 8 L/min at a constant temperature of 70 °C. The SEM images of nanowires on AAO templates at each flow rate under different magnifications are shown in Figures S2–S10, and a set of representative images at selected flow rates is shown in Figure 3a–e. The growth area ratio, which is the ratio between the area that has nanowires and the area of the AAO template used in a single growth process, is plotted in Figure 3f. More detailed data on the growth area and growth area ratio are shown in Table S1. Due to the dynamics of nanowire growth, as discussed above, not every sample can successfully produce nanowires even under the same growth conditions. As a result, the data points shown in the figure and the table are representative of the best sample obtained under the specified growth conditions.

Only large crystallites could be found when no gas flows through the growth chamber, and we observe large crystallites instead of nanowires, as shown in Figure 3a and Figure S2. When a steady flow of N_2_ was connected to the growth chamber, some free-standing nanowires could be observed. At low gas flow rates below 0.8 L/min, the evaporation of the solvent and the formation of nuclei were still limited by the removal of DMSO vapor, resulting in well-separated single nanowires, as shown in Figure 3b and Figures S3 and S5.

The largest areas of free-standing nanowires are found at a flow rate of 0.8 L/min. This flow rate also yielded a rather homogeneous length and density of nanowires, as shown in the low-magnification SEM images in Figure S6b,c. At gas flow rates higher than 0.8 L/min, the solvent evaporated more quickly, leading to an increase in the number of nuclei formed in the AAO templates and, thus, denser nanowire arrays. Note however that the growth area ratios are substantially smaller than that at 0.8 L/min, which can be seen in Figure 3f. Also, nanowires that grow too densely tend to aggregate and even merge, as shown in Figure 3d and Figures S7 and S8.

When the gas flow rate exceeded 4 L/min, the flow rate was so high that it strongly prohibited the growth of nanowires. Only a few sporadic nanowires were found, as shown in Figure 3e and Figures S9 and S10.

Next, nanowires grew under different temperatures ranging from 30 to 110 °C at a constant flow rate of 0.8 L/min. Representative SEM images are shown in Figure 4a–e, and more SEM images for other temperatures at different scales can be found in Figures S11–S19. The growth area ratio vs temperature is plotted in Figure 4f and listed in Table S2.

**Figure 4:**
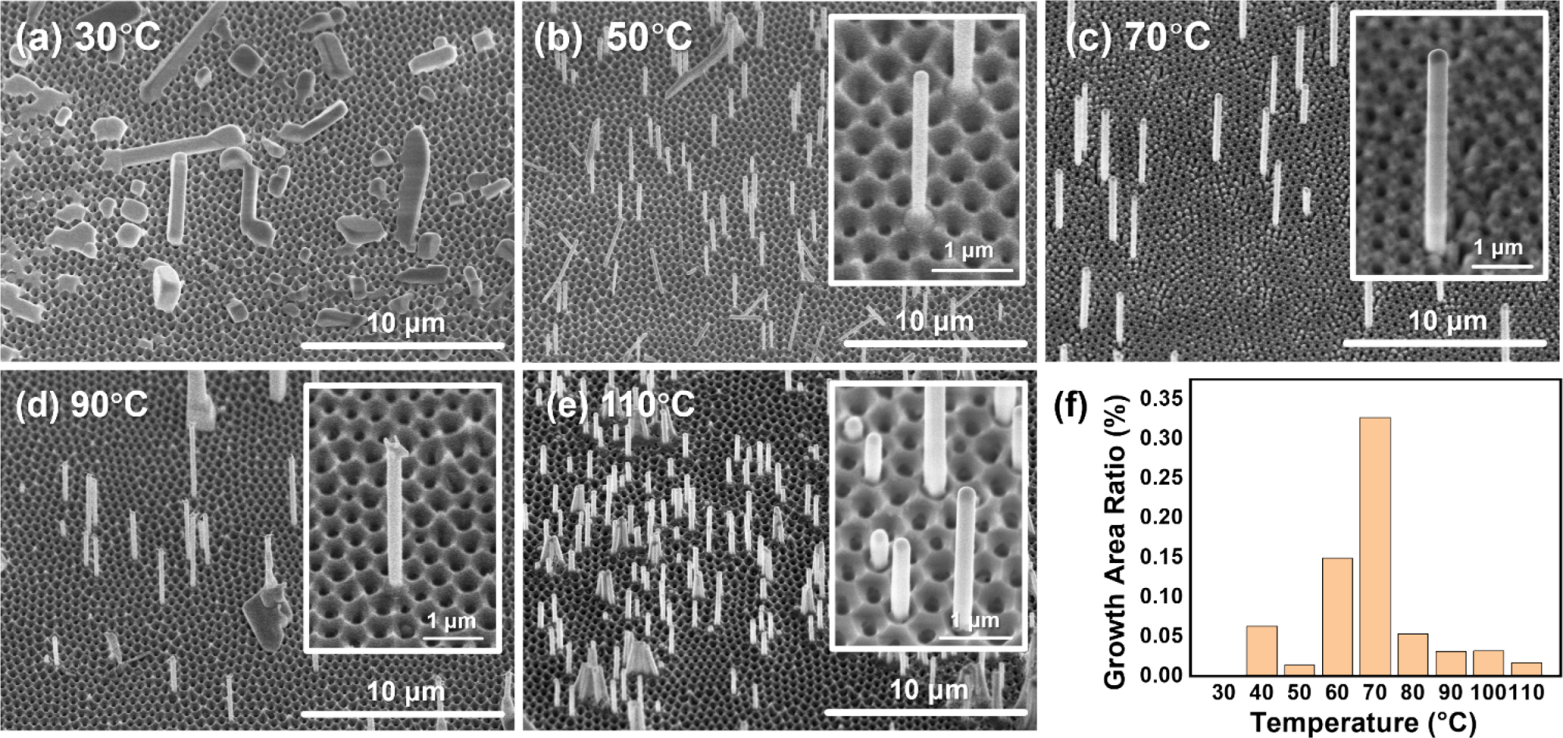
Temperature variation. SEM images of free-standing nanowires grew under (a) 30 °C, (b) 50 °C, (c) 70 °C, which is the same as Figure 3c, (d) 90 °C, and (e) 110 °C. The inset shows the high-resolution SEM image of single/several nanowires at each temperature. (f) Growth area ratio at different temperatures. The insets show high-resolution SEM images of single nanowires under each temperature. The SEM images are tilted by 30°.

No free-standing nanowires could be found when the growth temperature was lower than 40 °C, as shown in Figure 4a and Figure S11, similar to the growth at a zero gas flow rate. When the temperature exceeded 40 °C, increasingly large areas of nanowires could be observed. We find that 70 °C gave the best overall yield of nanowires. With further increase in temperature, the local density of nanowires increased, and aggregation of nanowires could be observed under high temperatures. However, we also found that these areas were smaller, leading to a significant decrease in the growth area ratio. We note that nanowires could be grown at temperatures significantly above the crystal phase transition from orthorhombic to tetragonal at 88 °C.^43^

Photoluminescence (PL) spectra and energy-dispersive X-ray spectroscopy (EDS) were conducted to further characterize the optical and chemical characteristics of the nanowires. The PL spectrum is shown in Figure S20. The Gaussian fit shows that the PL peak is at 521.5 nm with a full width at half-maximum (fwhm) of 22.5 nm, which is consistent with the values previously reported for CsPbBr_3_.^44−46^ The miscellaneous peaks at around 400 nm are related to the PL setup. The EDS spectrum shown in Figure S21 indicates a uniform distribution of Cs, Pb, and Br elements along the nanowire, and an average element ratio of ∼1:1.1:2.9 (Cs/Pb/Br) through the entire nanowire listed in Table S3 agrees closely with the stoichiometric ratio of CsPbBr_3_. The above analysis shows the high quality and purity of CsPbBr_3_ nanowires grown using such a method.

Overall, increasing the temperature had effects similar to those of increasing the flow, which is not unexpected. The diameter and overall length of nanowires are similar at different flow rates and temperatures, and mainly depend on the size of the pore in AAO templates and the volume of precursor solution used, respectively.^38^ In both flow rate and temperature studies, abnormal cases occurred, such as the sudden decrease in the nanowire growth area ratio at 50 °C and the rather large number of nanowires at 0.2 L/min, as shown in Figures 3f and 4f, respectively. These cases are tentatively attributed to fluctuations in local growth conditions inside a reactor. Despite these outliers, a clear optimum can be observed at a flow rate of 0.8 L/min and temperature of 70 °C. Under this optimized growth condition, we successfully grow free-standing nanowires in about 60% of batches, compared with approximately 17% without the flow reactor.

## Conclusions

In conclusion, we achieved a significant yield of free-standing CsPbBr_3_ nanowires grown in AAO templates by controlled evaporation crystallization. Slowing the crystallization by a controlled N_2_ flow and optimized heating in a custom-made reactor were found to be the keys to improved nanowire growth. The best growth parameters were identified as 70 °C reactor temperature with a flow rate of 0.8 L/min, leading to a batch success rate of 60%. *In situ* microscopy revealed a dynamic growth process that proceeds quickly and locally. The regions of the AAO membrane with free-standing nanowires exhibited pronounced photoluminescence during the initial stages of growth. To further improve the yield of nanowire growth, future studies could seek to imitate the sudden influx of N_2_ to quickly reach supersaturation and investigate the correlation of enhanced photoluminescence with the outcome of crystallization. The improved nanowire yield and control over the growth process provide a solid foundation for future integration of free-standing MHP nanowires into devices.

## Supporting Information Available

Schematic diagram of the growth setup; detailed tables of the growth area ratio under different conditions; SEM images under different growth conditions (PDF)

## ABBREVIATIONS

AAO: anodized aluminum oxide
DMSO: dimethyl sulfoxide
EDS: energy-dispersive X-ray spectroscopy
fwhm: full width at half-maximum
LEDs: light emitting diodes
MHP: metal halide perovskite
PL: photoluminescence
SEM: scanning electron microscopy
UV: ultraviolet
