# Supplementary material for: Controlled Ligand-Free Growth of Free-Standing CsPbBr3 Perovskite Nanowires
Source: ACS Omega. 2024 Nov 26;9(49):48390–6. doi: 10.1021/acsomega.4c06646 (PMC11635498; doi:10.1021/acsomega.4c06646)
Supplement: Supplementary file 1 — ao4c06646_si_001.pdf [file ao4c06646_si_001.pdf]

SUPPORTING INFORMATION

Controlled Ligand-Free Growth of Free-Standing $CsPbBr_3$ Perov-skite Nanowires

Ziyun Huang[1*], Zhaojun Zhang[1], Nils Lamers[1], Dmitry Baranov[2], Jesper Wallentin[1]

[1] Synchrotron Radiation Research and NanoLund, Department of Physics, Lund University,

Box 124, Lund, 22100, Sweden.

[2] Division of Chemical Physics and NanoLund, Department of Chemistry, Lund University, Box 124,

Lund, 22100, Sweden.

Corresponding author: ziyun.huang@sljus.lu.se

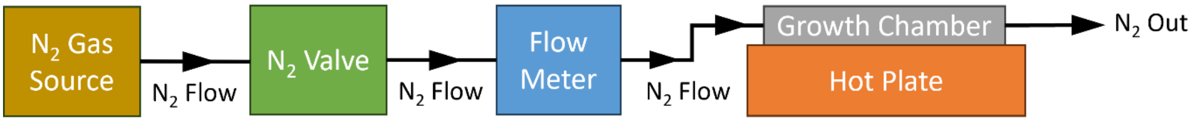

Figure S1. Schematic diagram of the growth setup.

Table S1. Growth area ratio at different flow rate under 70°C

**Temp (°C)**	**Flow Rate (L/min)**	**Area (μm^2)**	**Percentage (%)**
70	0	0	0
70	0.2	6.73×10^4	1.01×10^{-1}
70	0.4	4.63×10^3	6.70×10^{-3}
70	0.6	1.12×10^4	1.69×10^{-2}
70	0.8	2.17×10^5	3.27×10^{-1}
70	1	1.12×10^5	1.69×10^{-1}
70	2	3.36×10^4	5.07×10^{-2}
70	4	2.79×10^3	4.20×10^{-3}
70	8	2.83×10^3	4.26×10^{-3}

Table S2. Growth area ratio under different temperature at 0.8 L/min N_2 flow rate

**Temp (°C)**	**Flow Rate (L/min)**	**Area (μm^2)**	**Percentage (%)**
30	0.8	0	0
40	0.8	4.17×10^4	6.28×10^{-2}
50	0.8	9.16×10^3	1.38×10^{-2}
60	0.8	9.88×10^4	1.49×10^{-1}
70	0.8	2.17×10^5	3.27×10^{-1}
80	0.8	3.51×10^4	5.29×10^{-2}
90	0.8	2.06×10^4	3.11×10^{-2}
100	0.8	2.09×10^4	3.15×10^{-2}
110	0.8	1.11×10^4	1.68×10^{-2}

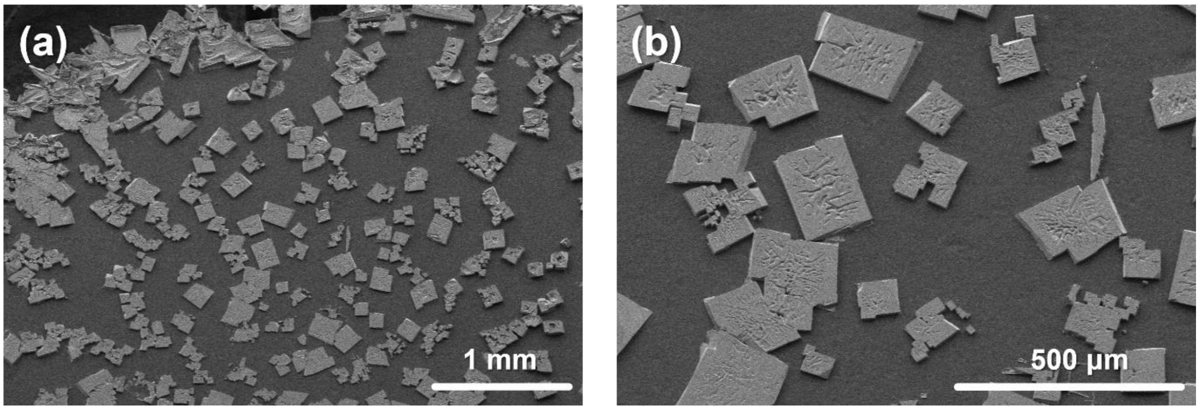

Figure S2. SEM images of free-standing nanowires in AAO substrate at (a) ×30 and (b) ×1.00k magnifications grew under 70°C without N_2 flow. The image is tilted by 30°.

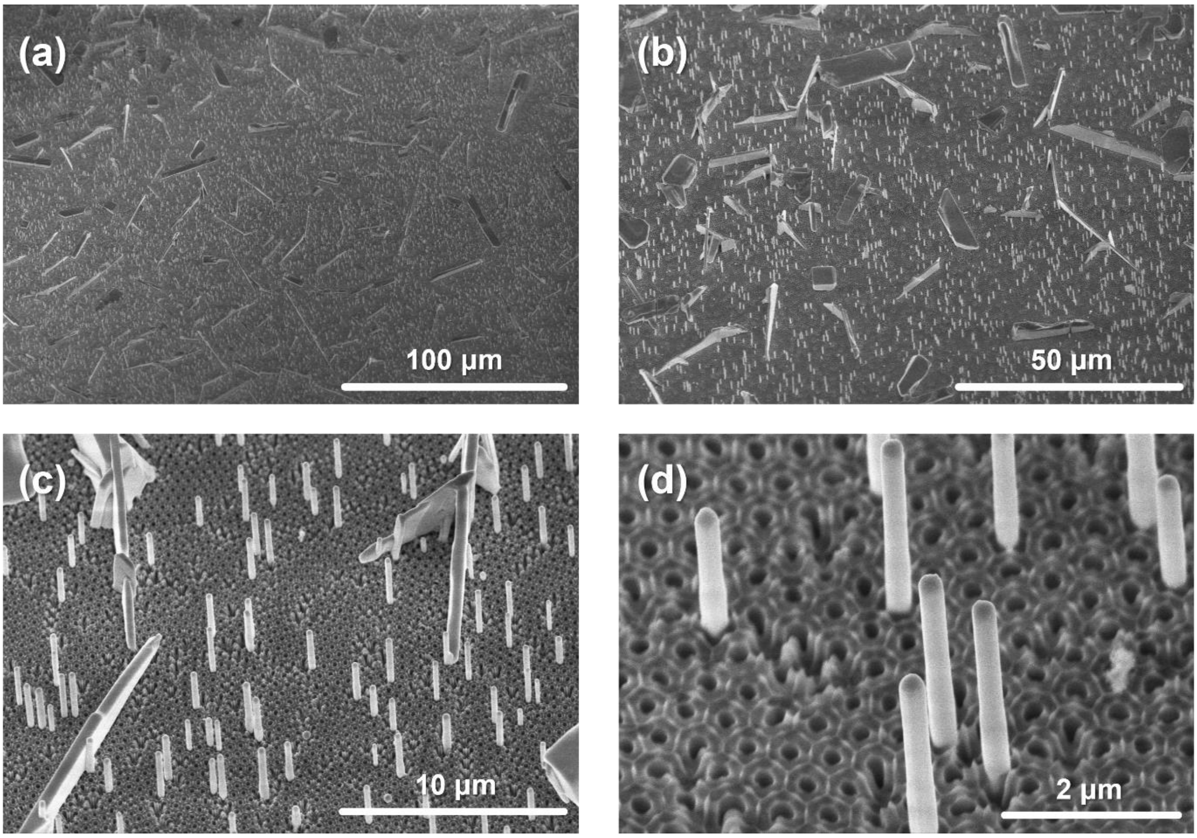

Figure S3. SEM images of free-standing nanowires at (a) ×500, (b) ×1k, (c) ×5k and (d) ×20k magnifications grew under 70°C at a flow rate of 0.2 L/M. The image is tilted by 30°.

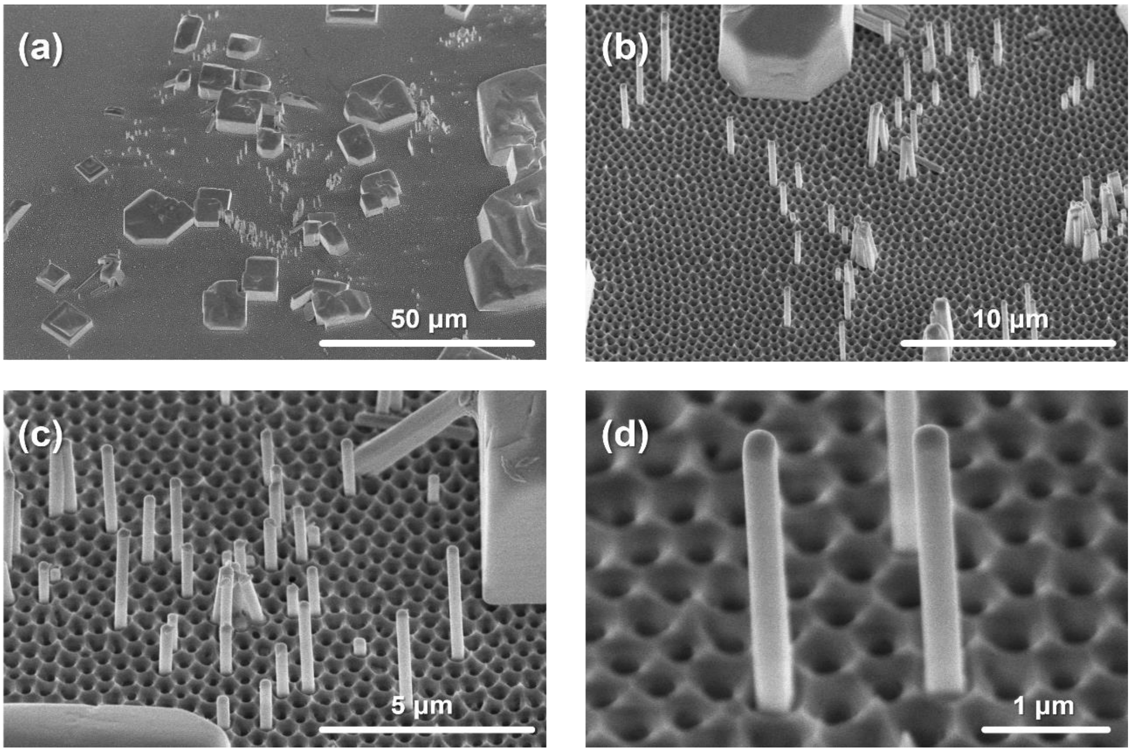

Figure S4. SEM images of free-standing nanowires at (a) ×1k, (b) ×5k, (c) ×10k and (d) × 50k magnifications grew under 70°C at a flow rate of 0.4 L/M. The image is tilted by 30°.

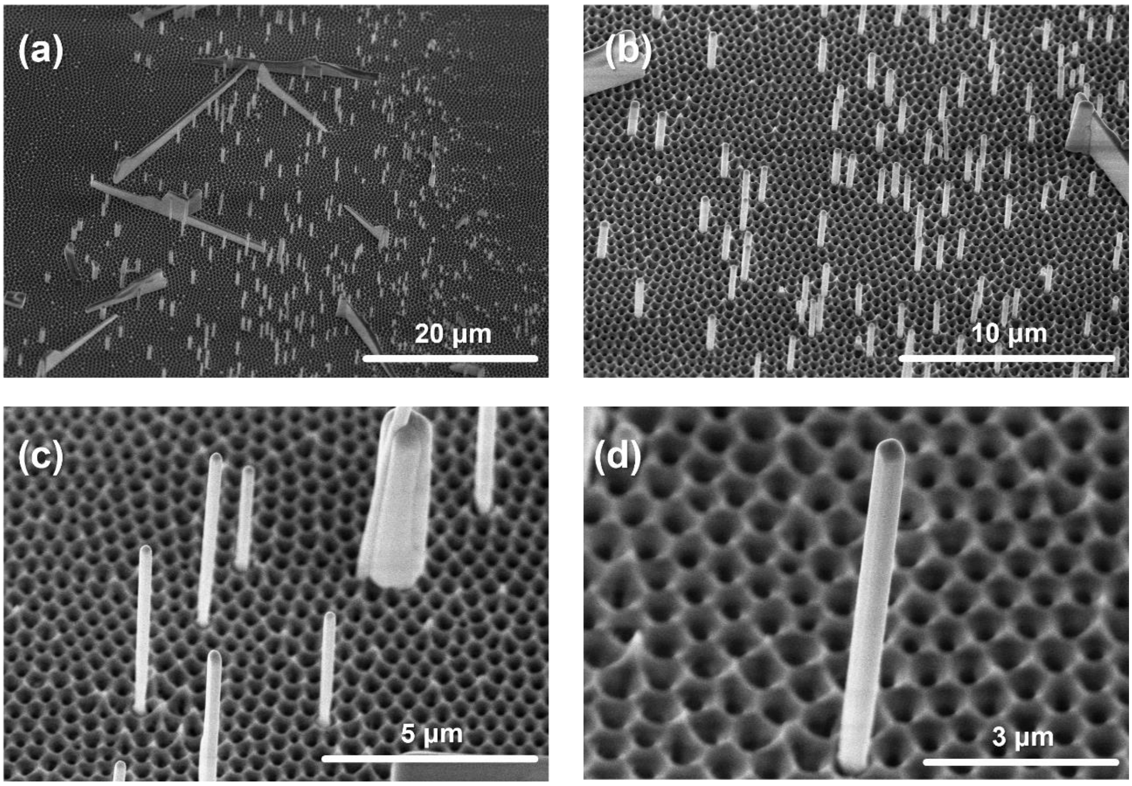

Figure S5. SEM images of free-standing nanowires at (a) ×2k, (b) ×5k, (c) ×10k and (d) × 18k magnifications grew under 70°C at a flow rate of 0.6 L/M. The image is tilted by 30°.

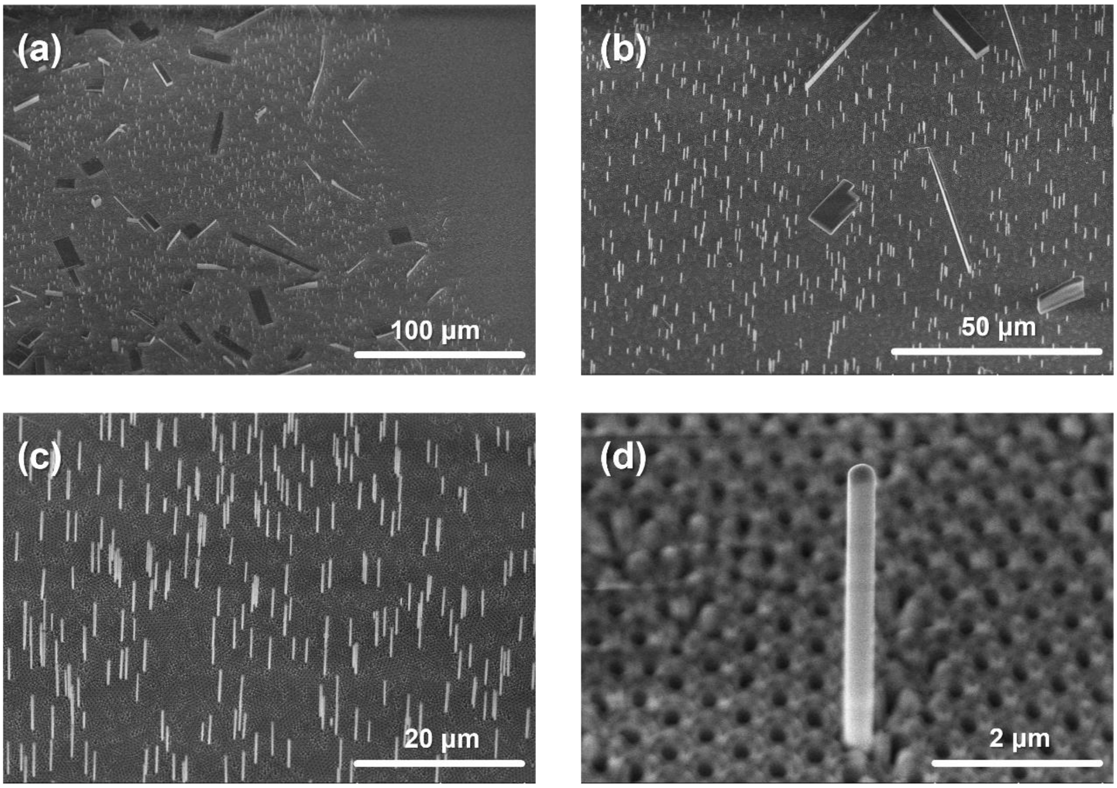

Figure S6. SEM images of free-standing nanowires at (a) ×500, (b) ×1k, (c) ×2k and (d) 20k magnifications grew under 70°C at a flow rate of 0.8 L/M. The image is tilted by 30°.

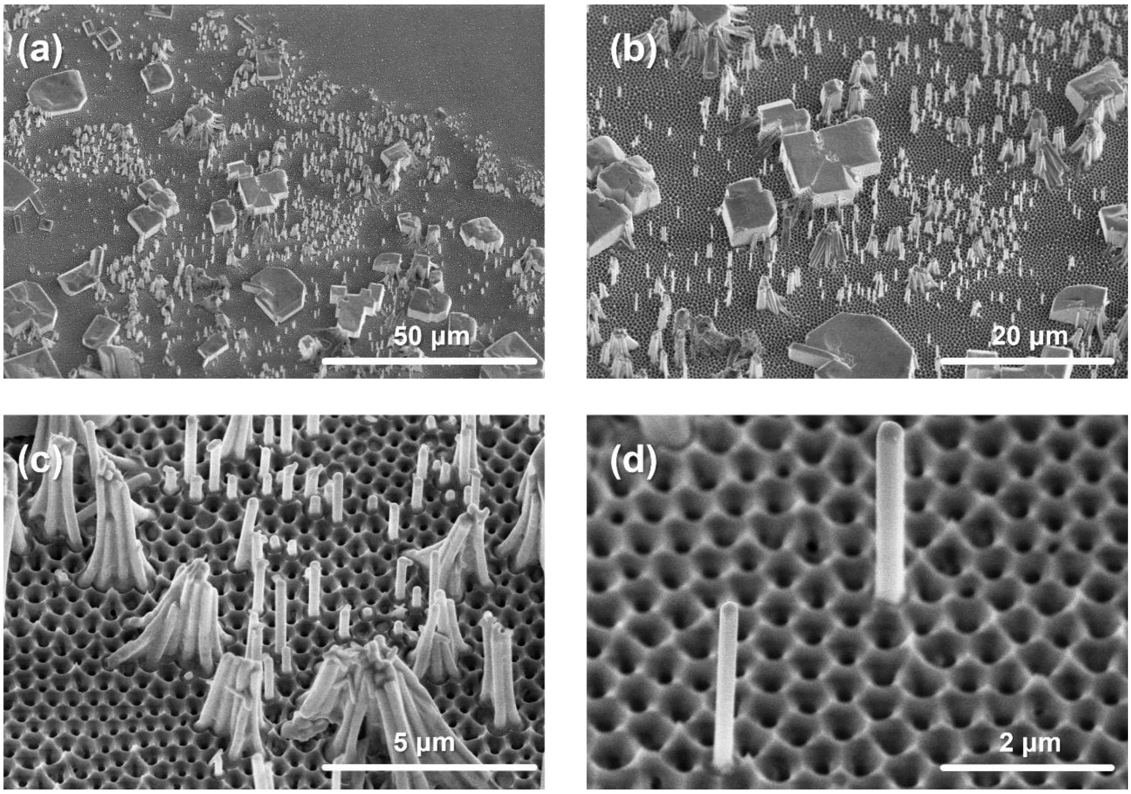

Figure S7. SEM images of free-standing nanowires at (a) ×1k, (b) ×2k, (c) ×10k and (d) × 20k magnifications grew under 70°C at a flow rate of 1.0 L/M. The image is tilted by 30°.

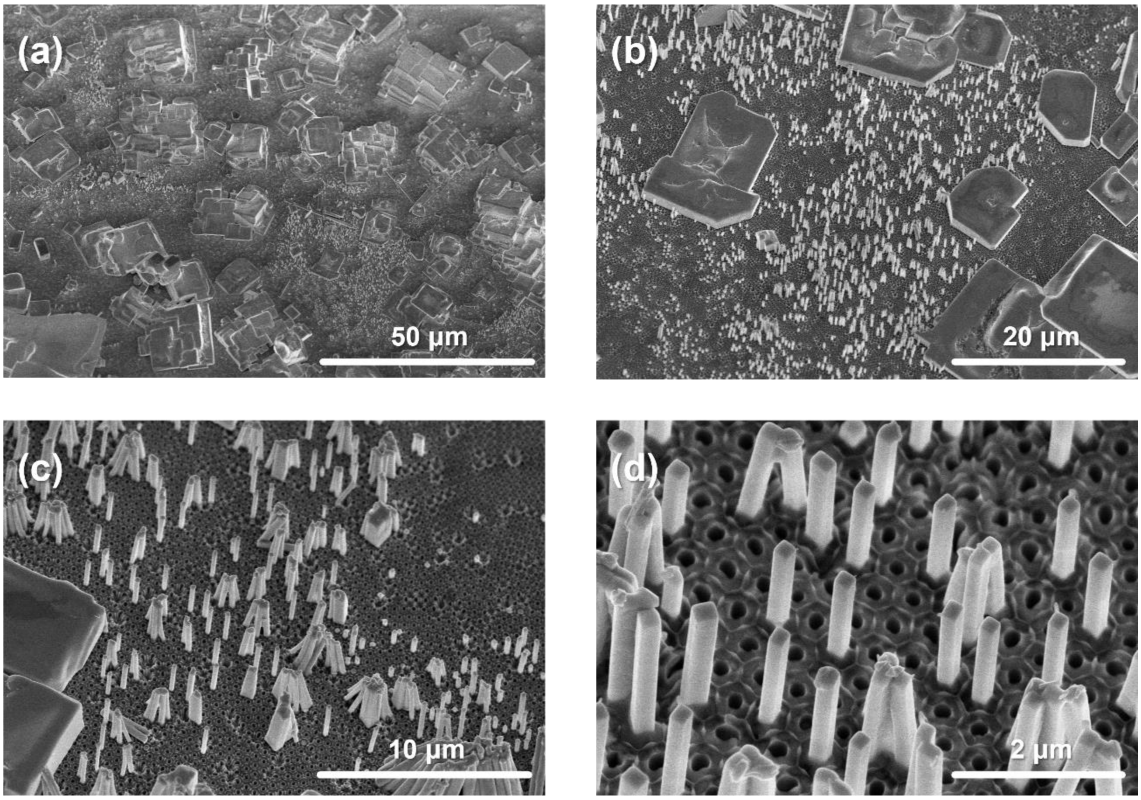

Figure S8. SEM images of free-standing nanowires at (a) ×1k, (b) ×2k, (c) ×5k and (d) ×20k magnifications grew under 70°C at a flow rate of 2.0 L/M. The image is tilted by 30°.

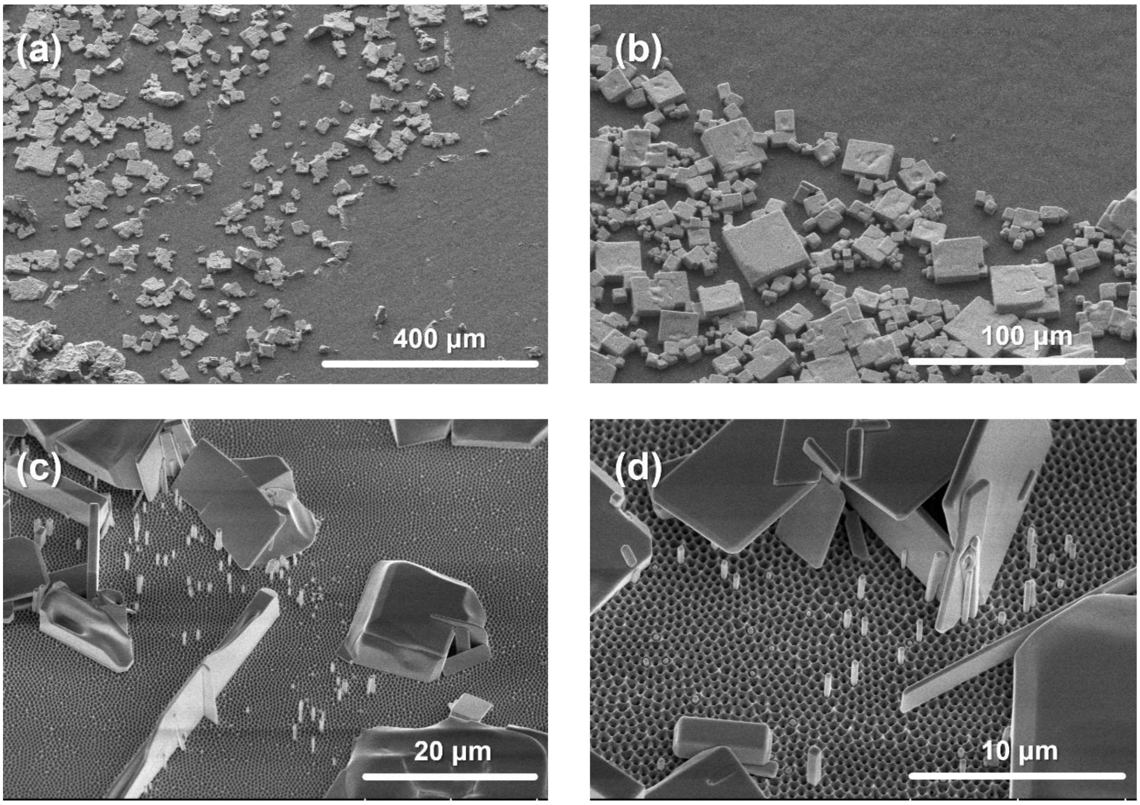

Figure S9. SEM images of free-standing nanowires at (a) ×120, (b) ×500, (c) ×2k and (d) × 5k magnifications grew under 70°C at a flow rate of 4.0 L/M. The image is tilted by 30°.

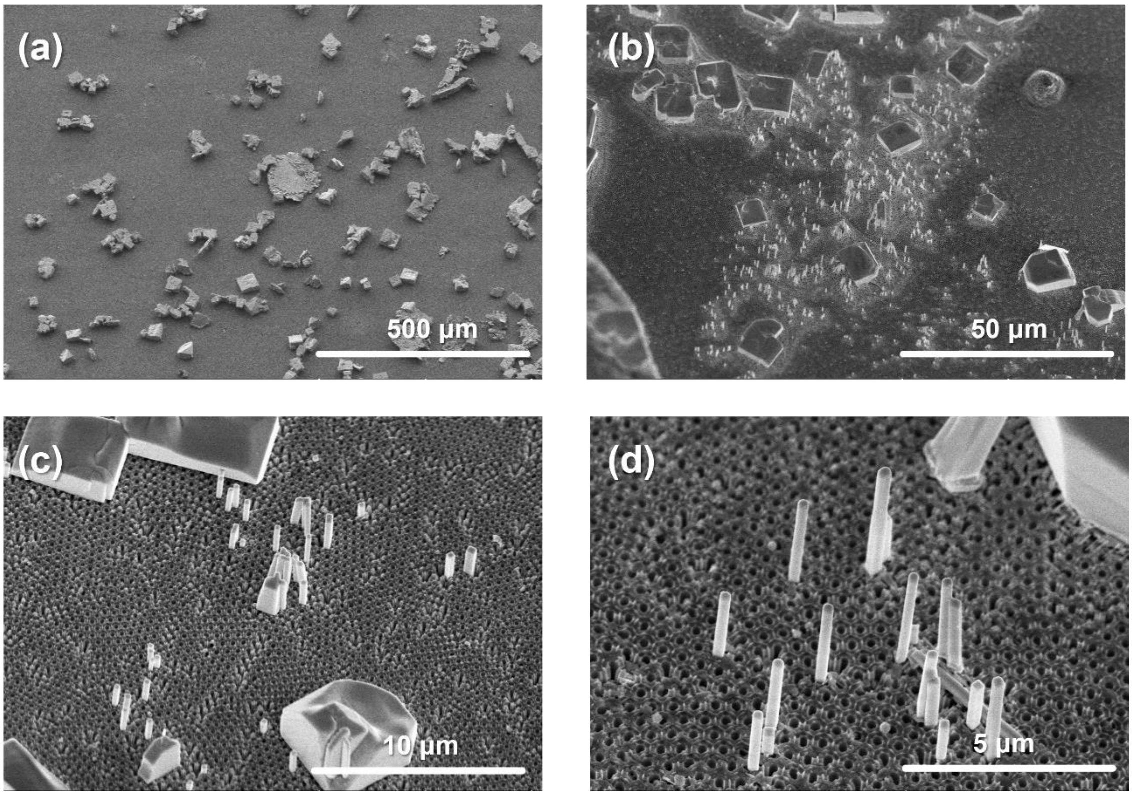

Figure S10. SEM images of free-standing nanowires at (a) ×100, (b) ×1k, (c) ×5k and (d) × 10k magnifications grew under 70°C at a flow rate of 8.0 L/M. The image is tilted by 30°.

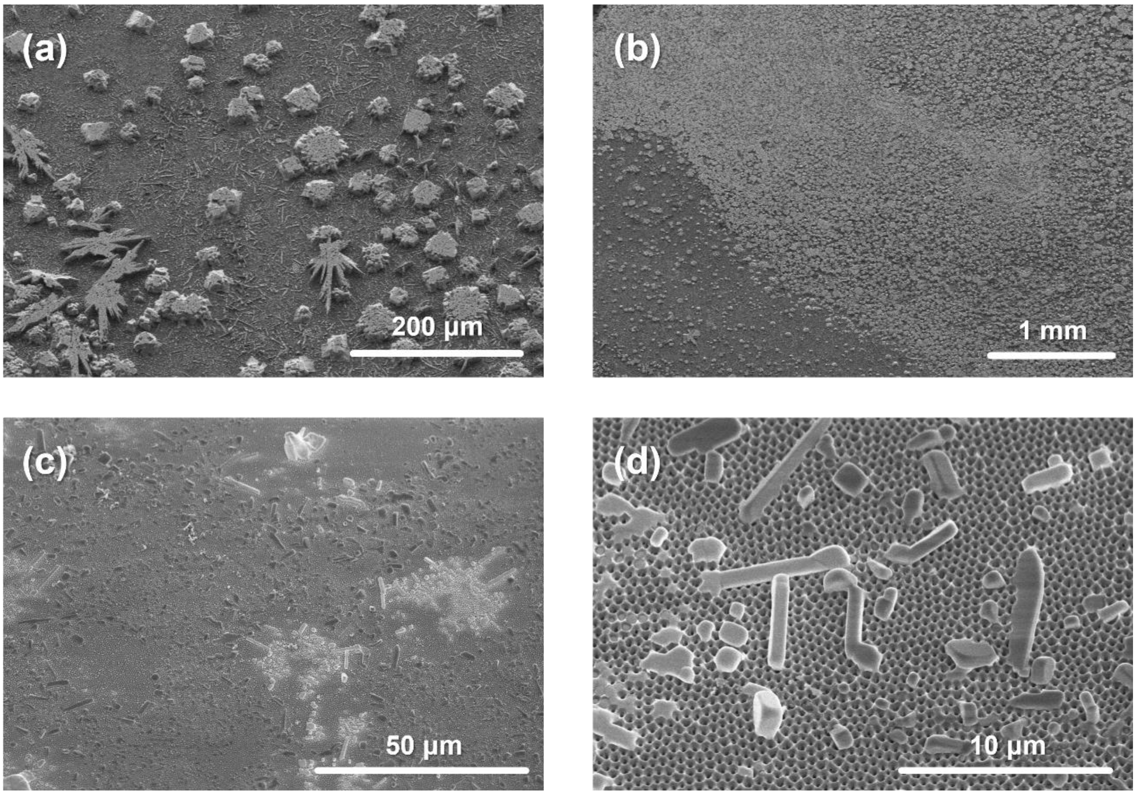

Figure S11. SEM images of free-standing nanowires at (a) ×200, (b) ×30, (c) ×1k and (d) × 5k magnifications grew under 30°C at a flow rate of 0.8L/M. The images are tilted by 30°.

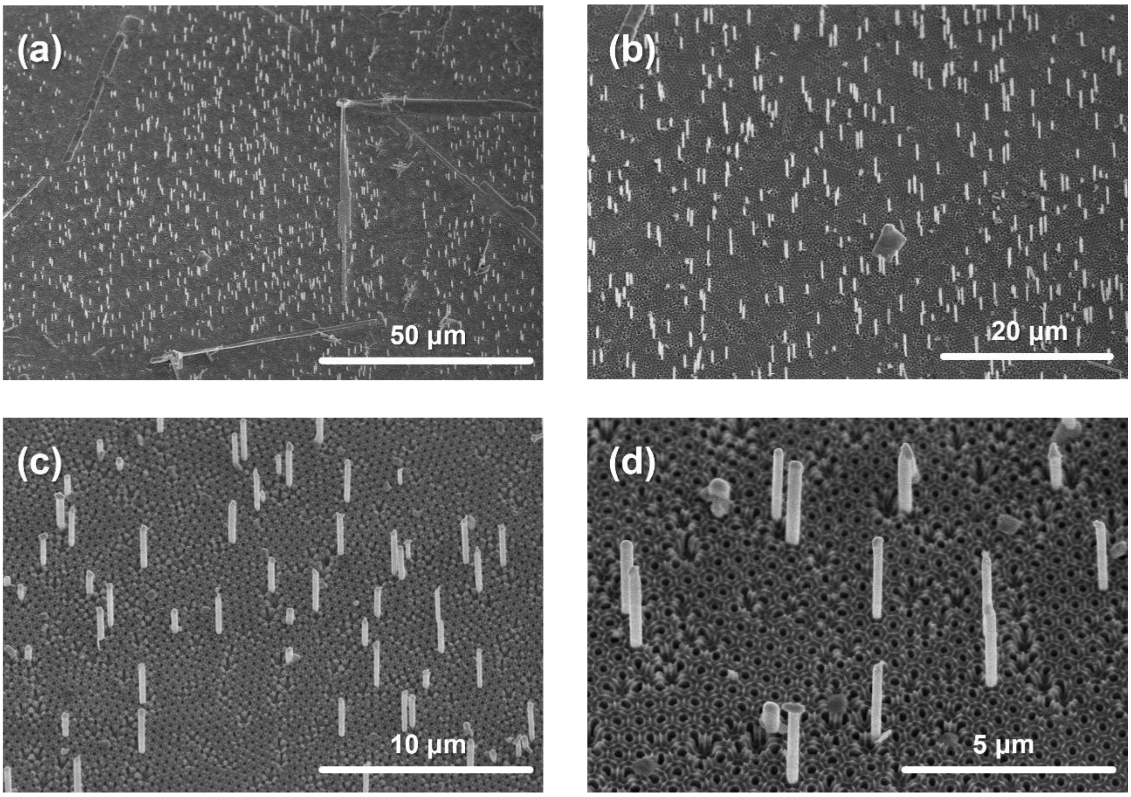

Figure S12. SEM images of nanowires at (a) ×1k, (b) ×2k, (c) ×5k and (d) ×10k magnifications grew under 40°C at a flow rate of 0.8L/M. The images are tilted by 30°.

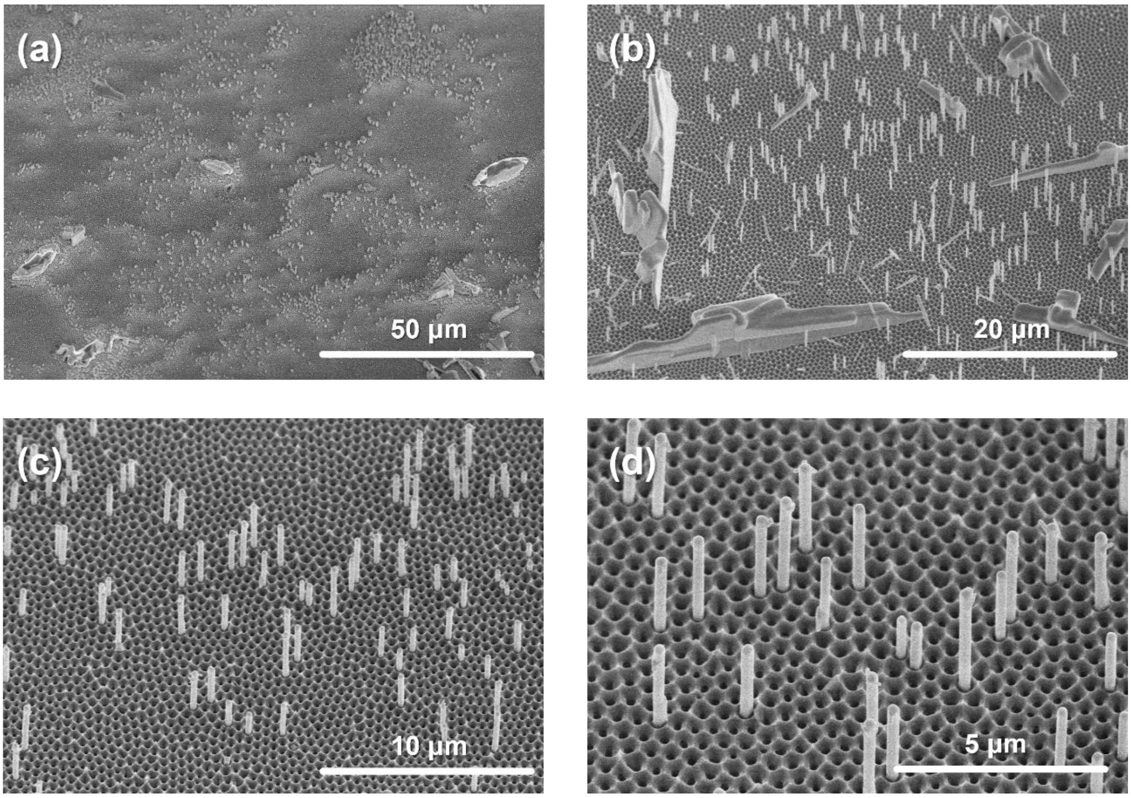

Figure S13. SEM images of free-standing nanowires at (a) ×1k, (b) ×2k, (c) ×5k and (d) × 10k magnifications grew under 50°C at a flow rate of 0.8L/M. The images are tilted by 30°.

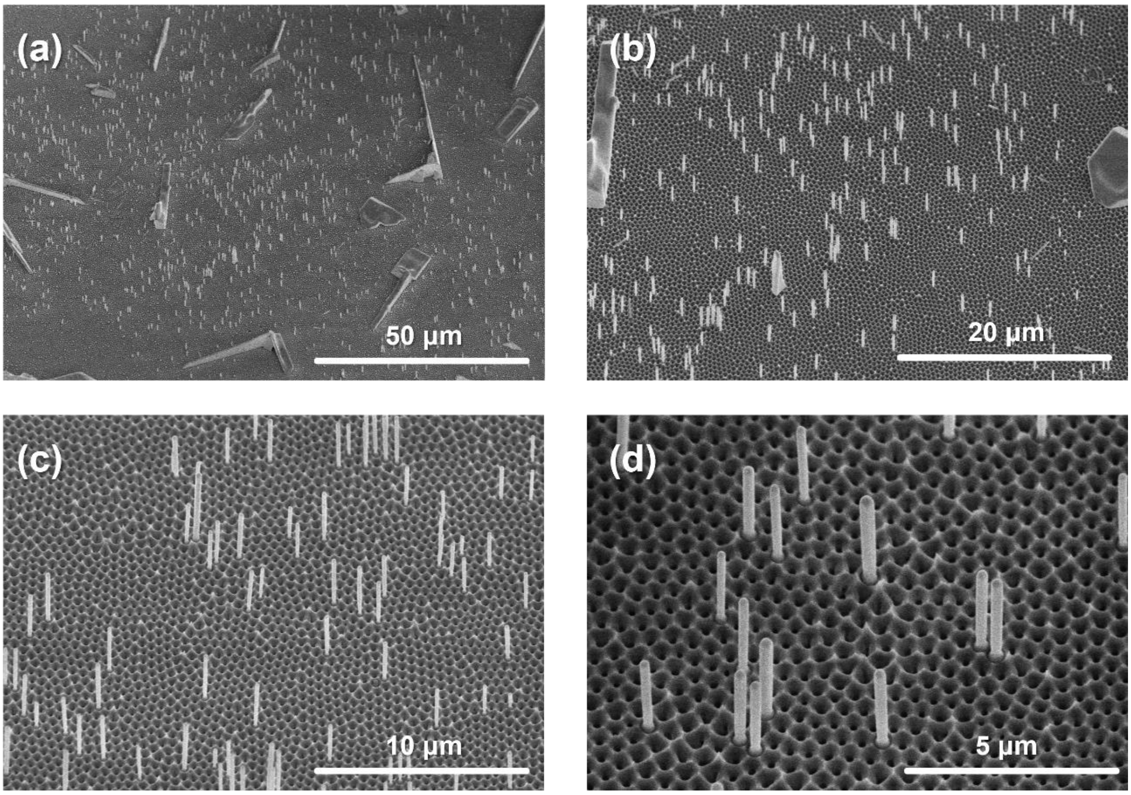

Figure S14. SEM images of free-standing nanowires at (a) ×1k, (b) ×2k, (c) ×5k and (d) × 10k magnifications grew under 60°C at a flow rate of 0.8L/M. The images are tilted by 30°.

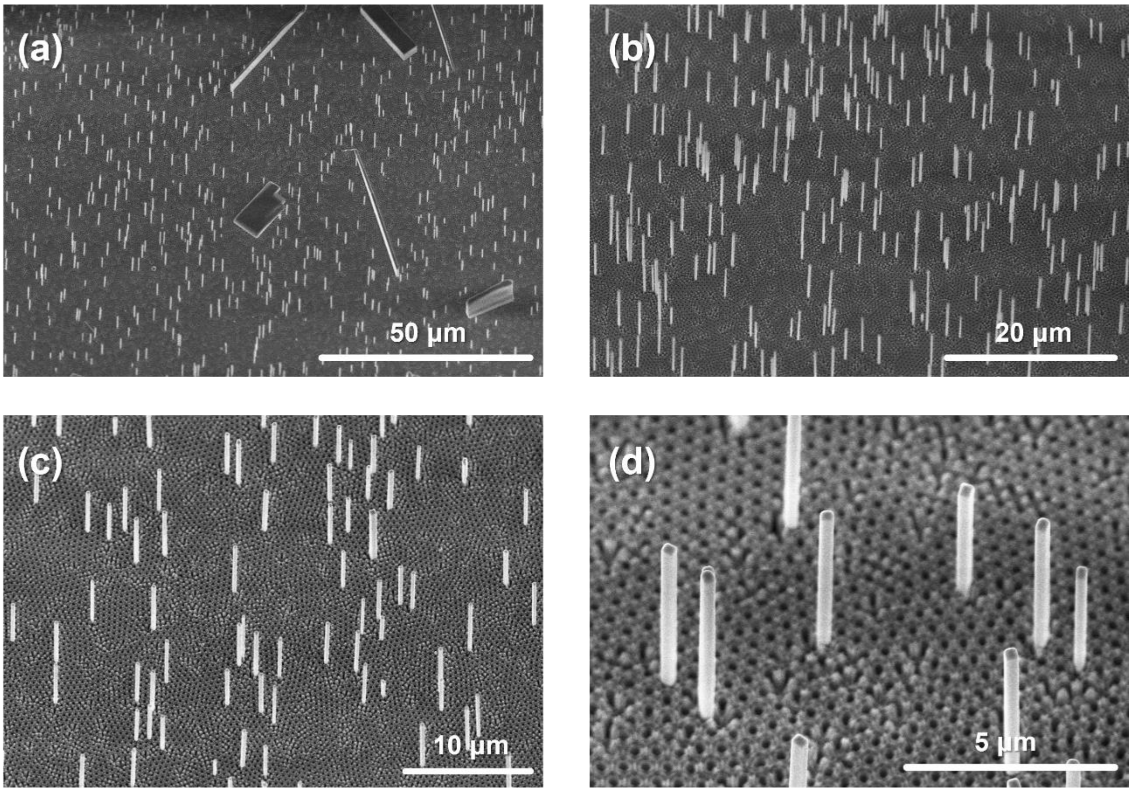

Figure S15. SEM images of free-standing nanowires at (a) ×1k, (b) ×2k, (c) ×5k and (d) × 10k magnifications grew under 70°C at a flow rate of 0.8L/M. The images are tilted by 30°.

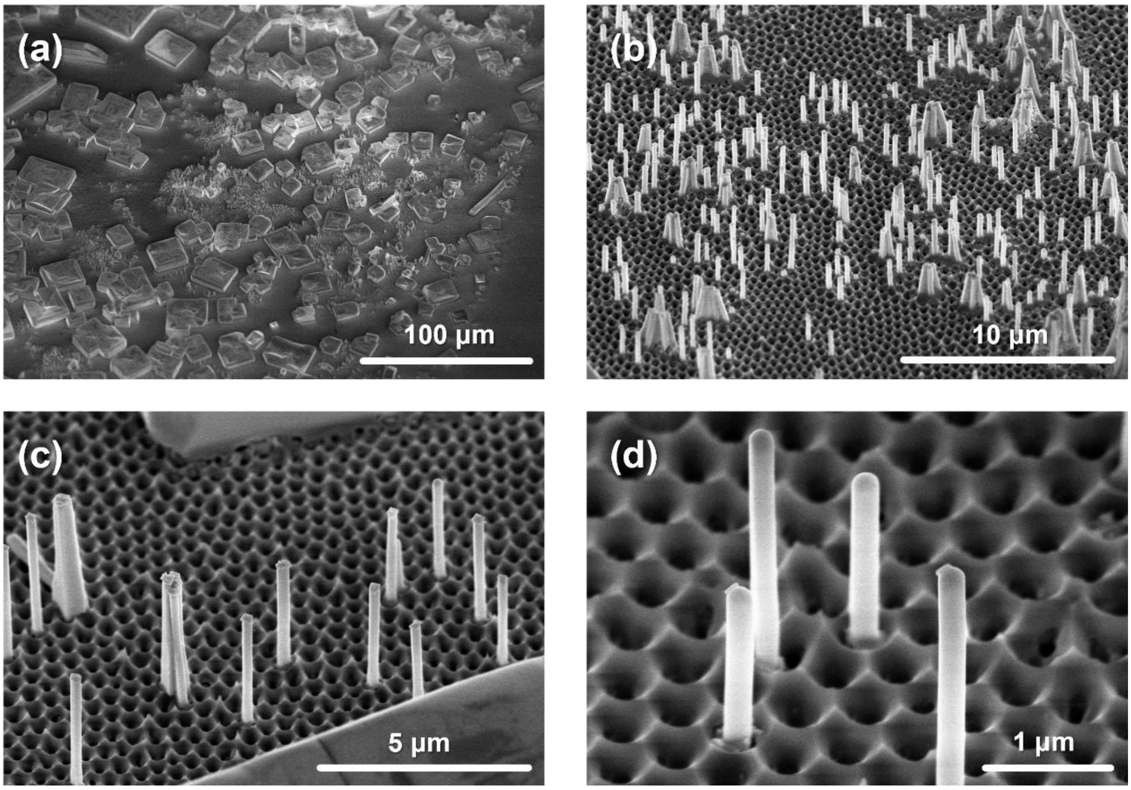

Figure S16. SEM images of nanowires grew under 80°C at a flow rate of 0.8L/M at (a) ×500, (b) ×5k, (c) ×10k and (d) ×50k magnifications. The images are tilted by 30°.

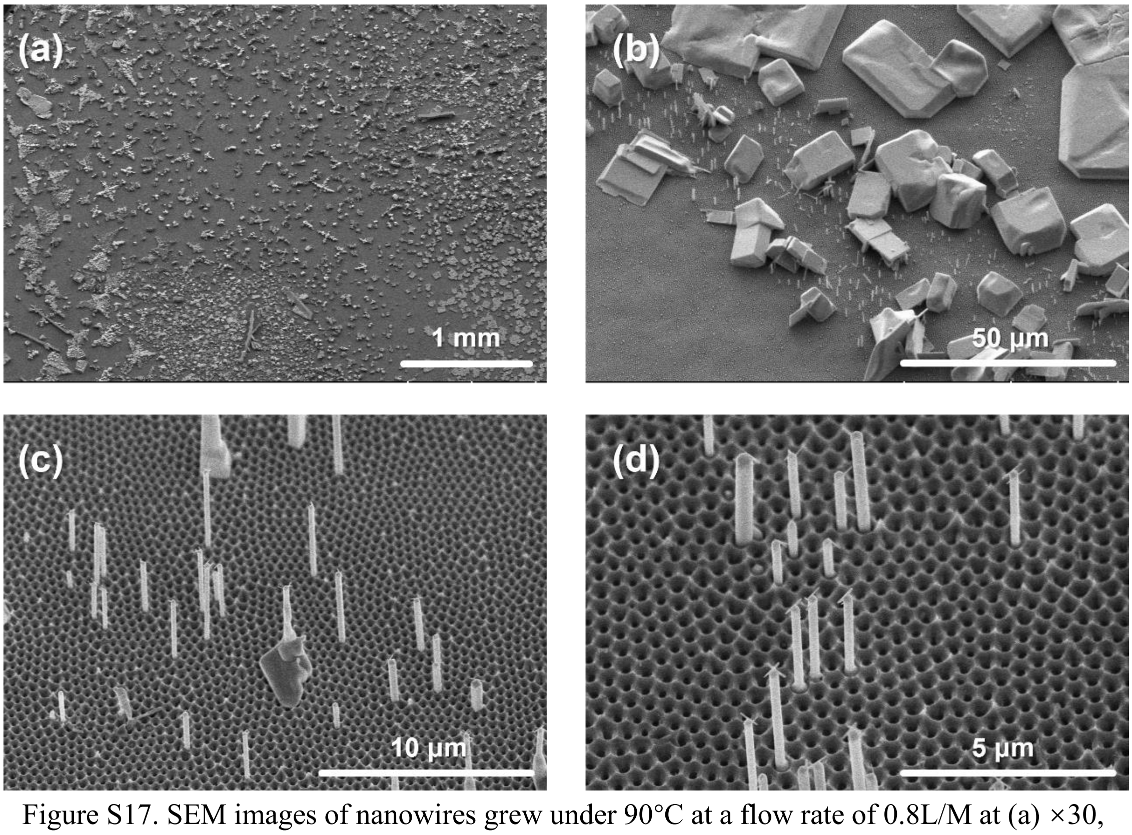

Figure S17. SEM images of nanowires grew under 90°C at a flow rate of 0.8L/M at (a) ×30, (b) ×1k, (c) ×5k and (d) ×10k magnifications. The images are tilted by 30°.

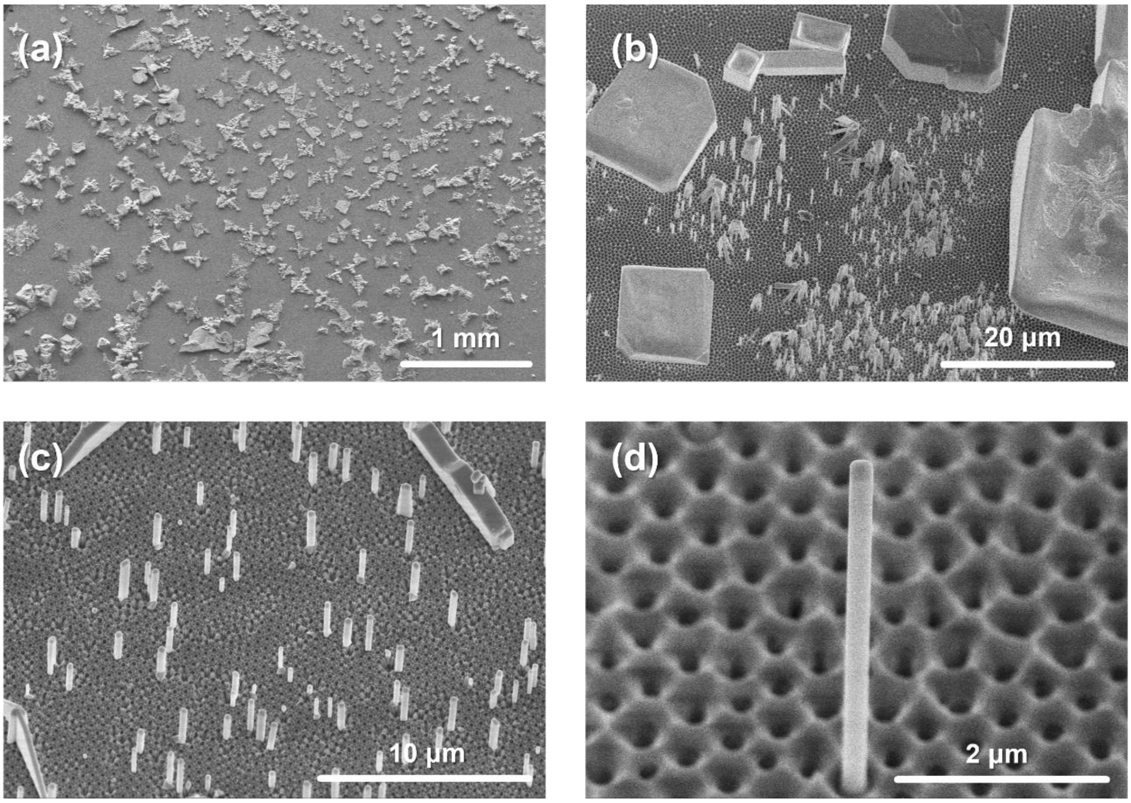

Figure S18. SEM images of nanowires grew under 100°C at a flow rate of 0.8L/M at (a) × 30, (b) ×3k, (c) ×5k and (d) ×20k magnifications. The images are tilted by 30°.

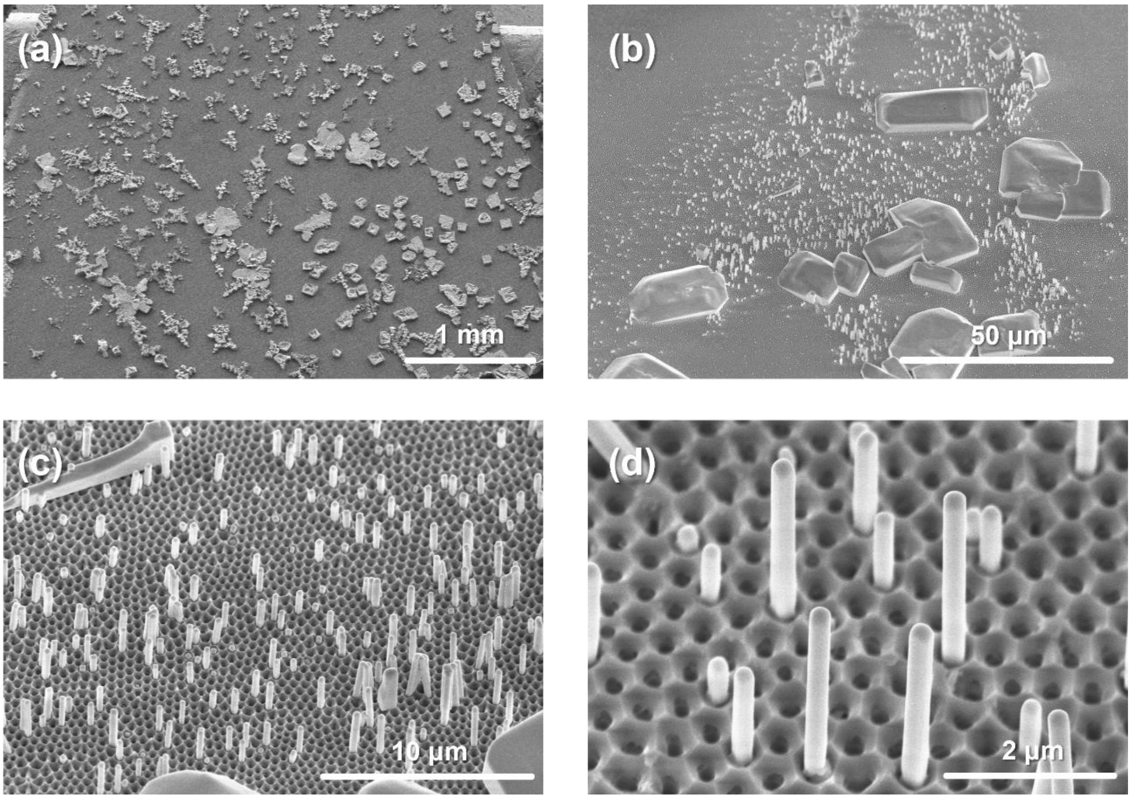

Figure S19. SEM images of nanowires grew under 110°C at a flow rate of 0.8L/M at (a) × 30, (b) ×1k, (c) ×5k and (d) ×20k magnifications. The images are tilted by 30°.

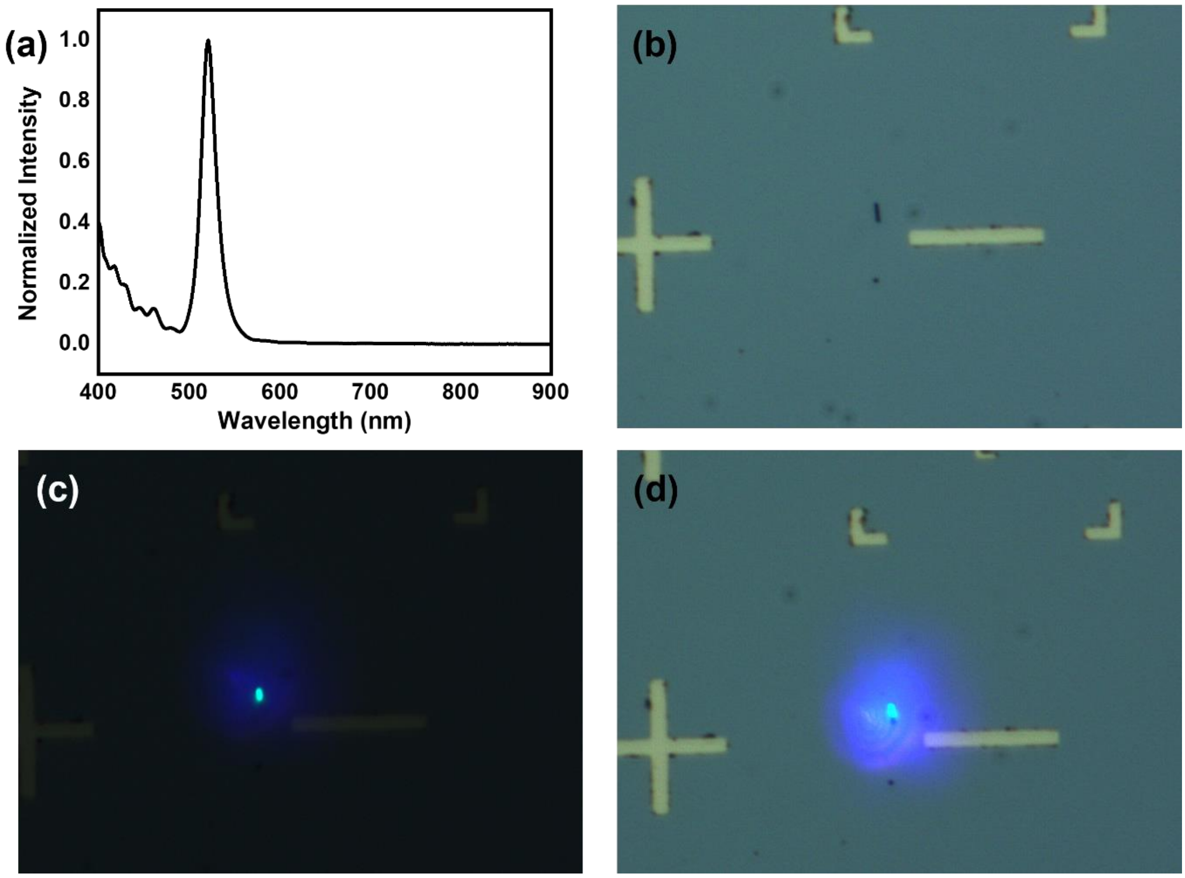

Figure S20. Photoluminescence (PL) of developed single nanowire on silicon substrate under 378 nm UV laser excitation (continuous-wave mode). (a) PL spectrum of a developed single nanowire. Optical microscope image of a single nanowire on silicon substrate. The PL peak of the nanowire is located at 521.5 nm, with FWHM of 22.5 nm. (b) Image under brightfield without excitation. (c) Image under dark field with excitation and (d) image under brightfield with excitation.

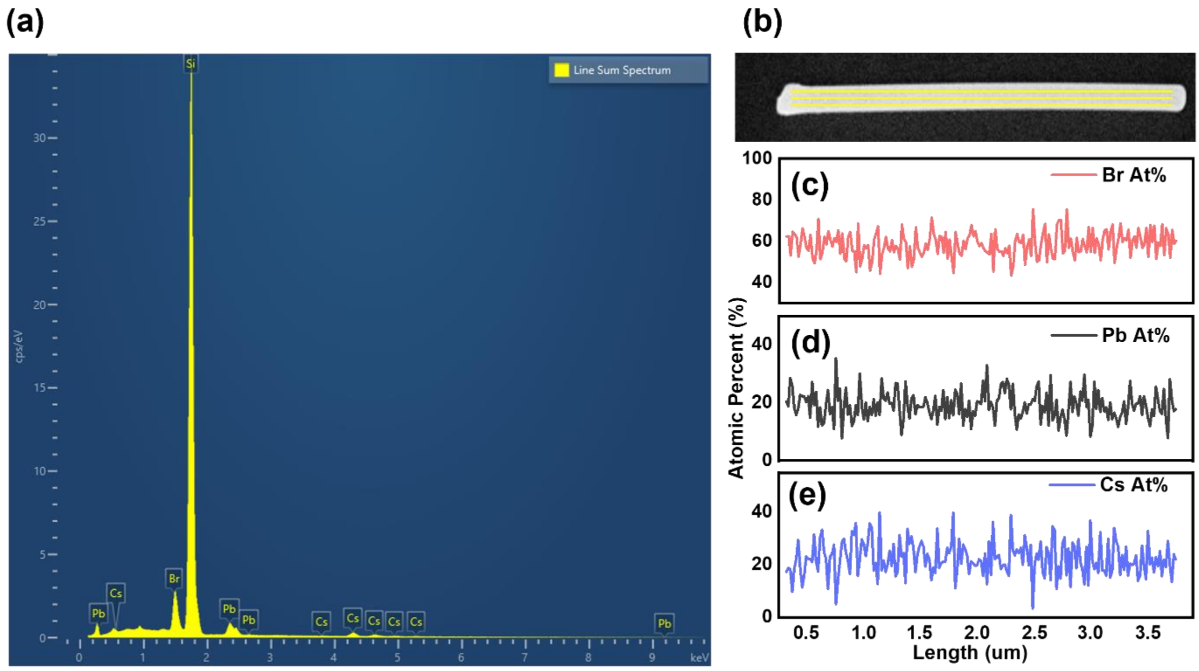

Figure S21. Energy-dispersive X-ray spectroscopy (EDS) of developed single nanowire on silicon substrate. (a) The EDS spectrum. (b) High resolution SEM image of the single nanowire. The three yellow lines indicates the EDS scan lines. The atomic percent of (c) Br, (d) Pb and (e) Cs along the scan lines.

Table S3. Average atomic percent of the nanowire

	Cs	Pb	Br
Atomic Percent (%)	19.16	22.18	58.65
